# Protocol for a systematic literature review on risk and protective factors for non-suicidal self-injurious behavior among adolescents in Iran

**DOI:** 10.1371/journal.pone.0359411

**Published:** 2026-09-28

**Authors:** Bita Erfanifar, Maud Modders, Karlijn Massar

**Affiliations:** 1 Work & Social Psychology, Faculty of Psychology & Neuroscience, Maastricht University, Maastricht, The Netherlands; 2 Department of Psychology, Islamic Azad University of Neyshabour, Iran; University of Botswana, BOTSWANA

## Abstract

**Background:**

Self-harm, including non-suicidal self-injury (NSSI), represents a major public health concern among adolescents. Although numerous risk and protective factors have been identified in the literature, their utility for assessment and management remains limited due to their large number and the variability in the strength of their associations. In Iran, in particular, there is a notable lack of synthesis, related to adolescent NSSI, especially regarding protective factors. To address this gap, this protocol outlines an ongoing systematic review aiming to provide a comprehensive overview of the recent (2015 onward) Persian and English peer-reviewed literature on NSSI and self-harm among adolescents in Iran, with a specific focus on studies investigating risk and protective factors.

**Methods & analysis:**

The protocol is registered with PROSPERO (registration number CRD420261286909), and the systematic literature review, following PRISMA-P guidelines, will include Persian and English-language studies from 2015 onwards examining risk and protective factors for non-suicidal self-injury (NSSI) in Iranian adolescents (10–19 years), as well as robustness of findings. Eligible studies include quantitative, qualitative, and mixed-methods designs, identified through PubMed, PsycINFO, Scopus, Embase, Google Scholar, SID, and Magiran. Data will be extracted using a standardized form, and study quality will be assessed with the Mixed Methods Assessment Tool (MMAT). Findings will be synthesized narratively, summarizing associations between risk or protective factors and NSSI, organized by clinically meaningful categories such as psychopathology, individual, family, peer, and demographic factors.

## Introduction

The World Health Organization [1] defines adolescence as the developmental stage between 10 and 19 years of age, a complex period characterized by profound biological, cognitive, behavioral, and emotional transformations, and a vulnerable stage of development marked by rapid hormonal, physical, and neural changes. During this period, many young people experience challenges related to mental well-being, with approximately one in five adolescents suffering from a psychiatric disorder that often continues into adulthood [2]. Girls and adolescents with lower perceived socioeconomic status are at greater risk for mental health issues, while the growing influence of peers in school settings increasingly shapes their behaviors and psychological well-being [3,4]. These multidimensional changes can substantially affect adolescents’ capacity to manage developmental challenges and interpersonal conflicts, thereby increasing the likelihood of maladaptive or risk-prone behaviors [5].

One maladaptive behavior that is typically more prevalent among adolescents than adults ([6,7] is non-suicidal self-injury (NSSI) which includes behaviors such as cutting, burning, and self-hitting. Importantly, NSSI is distinct from suicidal self-harm and other maladaptive behaviors such as disordered eating or social withdrawal [8]. In the current research, we utilize the term NSSI to refer to intentional self-injury without suicidal intent, while self-harm denotes a broader category including both suicidal and non-suicidal behaviors, including self-poisoning [9].

NSSI behaviors affect approximately 15–30% of school-aged youth, particularly those aged 12–14 years [10–12], and girls engage in NSSI more frequently than boys [13]. Adolescents engaging in NSSI behaviors differ in injury type, frequency, and underlying causes, and compared to peers with additional behavioral problems, those exhibiting only NSSI often show greater impairments in self-functioning [14,15]. In addition, NSSI has been linked to serious psychological consequences, including an elevated risk of suicide attempts [16,17], guilt, shame, and recurring negative emotions [18,19]. NSSI behaviors are often associated with substance use, childhood trauma, poor emotional regulation, and co-occurring disorders like borderline personality and eating disorders [10,20]. NSSI thus represents a significant mental health concern due to its psychological and social impacts, concealment, and role as a maladaptive coping strategy, and it is a strong predictor of suicide, highlighting the need for insight into both risk and protective factors to inform prevention and intervention [21,22].

### The context of Iran

In recent years, there has been increasing concern about the erosion of youth mental health in Iran. Socioeconomic strain, academic pressure, limited access to mental-health services, and rapid sociocultural change have been identified as important challenges to the mental health of Iranian youth [23]. Adolescent vulnerabilities for NSSI, such as psychological distress and difficulties in emotion regulation, likely operate similarly across cultures, yet the conditions under which these vulnerabilities emerge may be context-dependent. In Iran, family dynamics involving intergenerational relationships, parental psychological control, gendered expectations, and tensions surrounding adolescent autonomy may further shape how psychological distress develops and is expressed [24,25].

Thus, although the proximal psychological vulnerabilities underlying NSSI may not be unique to Iran, their sociocultural antecedents and relative importance may differ. For example, in the Iranian context, cultural attitudes and stigma surrounding mental health difficulties may also influence whether and how psychological distress and NSSI are disclosed, and whether professional support is sought [26]. The COVID-19 pandemic may have further impacted the risk and protective factors relevant to NSSI. International evidence suggests that adolescent self-harm and suicidality increased during the pandemic, potentially due to factors such as social isolation and increased family and financial stress [27]. Among Iranian adolescents, loneliness was associated with poorer well-being during the pandemic, whereas resilience and parental involvement were associated with better well-being [28]. Thus, identifying risk and protective factors in this changing social and cultural context is essential for informing appropriate prevention and intervention efforts for Iranian adolescents.

Given that NSSI is often concealed, and help-seeking remains limited, prevalence estimates are likely underestimated. Moreover, reported prevalence rates are frequently derived from studies on self-harm behaviors in general, rather than specifically on NSSI, and they focus predominantly on high-income or Western countries, with very few studies conducted in low- and middle-income or non-Western settings [29]. Indeed, the (reported) prevalence of NSSI varies considerably across regions; for instance, in Western countries such as the United States and Canada, lifetime prevalence estimates range from 1.5% to 54.8% [7]. Recognizing this bias helps to call for more inclusive, globally representative research, so that findings are more robust across diverse adolescent populations. In 2015, self-injury ranked as the third leading cause of adolescent mortality worldwide and the second in Europe and South-East Asia, while it was the eighth leading cause of death in the United States [30]. Despite its global relevance, research on NSSI in Iran remains limited. For example, Gholamzadeh et al. reported that 8.7% of individuals referred to forensic centers in Shiraz had engaged in self-injury [31].

### Risk and protective factors

Adolescents with mood, anxiety, or other psychiatric disorders are at a significantly elevated risk of engaging in NSSI, as these conditions heighten emotional distress and impair the development of adaptive coping strategies [32]. Furthermore, family-related factors such as conflict, low parental support, and insecure attachment have been shown to increase vulnerability to NSSI by contributing to psychological distress and constraining adolescents’ coping resources [33]. Although research on non-suicidal self-injury (NSSI) across diverse contexts remains in its early stages, it is increasingly evident that understanding the context-specific factors associated with NSSI is essential for developing culturally and locally appropriate interventions [34]. Evidence indicates that certain risk factors are more prevalent in specific contexts [35]. A systematic review conducted in low- and middle-income countries identified key risk factors for self-injury, including low paternal education, poor perceived family economic status, peer influence (self-injury by close friends or peer groups), low academic performance, and frequent school absenteeism [34]. Moreover, participants with a history of self-injury reported lower levels of social support than those without such behaviors [36].

In the context of Iran, several factors have been identified as increasing the likelihood of non-suicidal self-injury (NSSI) among adolescents, such as older age, cigarette smoking, sport team membership, involvement in physical fights, general risk-taking behaviors, and lower self-esteem [37]. Also, in another study, NSSI was associated with weak family psychological functioning and low perceived social support [38]. However, the literature on protective factors for Iranian youth that could ultimately be targeted in efforts to prevent or reduce NSSI, is much smaller. A recent study [39] showed that self-compassion mediates the relationship between family functioning and NSSI. Self-compassion has also shown to moderate the relationship between depressive symptoms and NSSI [40]. Furthermore, having a sense of meaning in life [41], a problem-oriented coping style [42], and psychological resilience [43] are among the variables investigated.

### Review aim

Previous reviews and review protocols have addressed NSSI in Iran, but these focused mainly on broader populations, prevalence of NSSI, or psychosocial correlates [44–46]. In the current systematic review, we will include and critically appraise both English- and Persian-language evidence. Moreover, in considering individual, family, social, and broader contextual factors, we aim to identify not only vulnerabilities associated with NSSI, but also potentially modifiable protective factors.

The aim of the current review therefore is to provide a comprehensive overview of the recent (2015 onward) peer-reviewed literature published in Persian or English on adolescent NSSI in Iran, with a particular focus on risk and protective factors. Specifically, we aim to answer the following questions:

- Which risk factors are associated with NSSI among Iranian adolescents?
- Which protective factors buffer against NSSI in this population?
- How consistent and robust is the evidence across study designs?

## Methods

This systematic review protocol follows the Preferred Reporting Items for Systematic Review and Meta-Analysis Protocols (PRISMA-P) guidelines [47]. The completed PRISMA-P checklist is provided in the Supporting Information (S1 File). The protocol is registered with PROSPERO (registration number CRD420261286909).

### Eligibility criteria

We utilized the PEO (Population, Exposure, Outcome) framework to develop the research question and the eligibility criteria for this review:

#### Study population.

Our target population consists of adolescents (i.e., individuals aged 10–19 years [1], or those defined as “adolescents” by study authors). The population must fall within this age range at the time of both exposure to the risk or protective factor and the outcome (only non-suicidal self-injury (NSSI)). Only studies conducted among the specified population in Iran, whether in school, community, or clinical settings, will be included. Multinational studies will also be eligible if they report separately extractable data for adolescents in Iran.

If a study includes both adolescents (10–19 years) and adults (over 20 years), it will be included only if (1) the average age of participants is under 20 years, (2) more than 50% of participants are adolescents, or (3) the study provides a subgroup analysis limited to adolescents.

#### Exposure and outcome variables.

Eligible studies must investigate the association between at least one risk or protective factor and non-suicidal self-injury (NSSI), with NSSI assessed as the primary outcome. NSSI is defined as the intentional, self-inflicted damage to body tissue without suicidal intent [48]. Studies focusing on suicidal behaviors, self-injury with intent to die, or those that do not examine relevant risk or protective factors will be excluded.

#### Types of studies to be included.

This review will include empirical studies that provide original data on non-suicidal self-injury (NSSI) in adolescents. Eligible study designs include cohort, case–control, cross-sectional, longitudinal, randomized controlled trials, quasi-experimental studies, qualitative research, and mixed-methods studies. Studies that do not report original empirical findings (e.g., case reports, editorials, commentaries, and patents) will be excluded. Systematic reviews and meta-analyses will also be excluded to avoid duplication of data. Included studies must examine risk factors, protective factors, or both in relation to NSSI. Articles published in Persian (Farsi) and English will be considered. Preliminary scoping revealed substantial variability in self-harm terminology in Persian-language sources, particularly regarding distinctions between NSSI, suicidal behavior, and broader self-harm. To maintain conceptual clarity and minimize the risk of misclassification, eligibility will therefore be determined based on the operational definition of the behavior examined rather than the terminology used. Studies will only be included when the reported behavior can be clearly identified as intentional self-injury without suicidal intent, consistent with the definition of NSSI adopted in this review.

### Search methods

The literature search will be conducted using the following international databases: PubMed, PsycINFO (via EBSCOhost), Scopus (via Elsevier), Embase (via OVID), Google Scholar, and the Iranian databases Scientific Information Database (SID) and Magiran. Searches will include studies published from January 2015 onward; studies published before 2015 will be excluded. The initial search was conducted on 5 December 2025 and will be updated monthly until submission of the final manuscript to ensure inclusion of the most recent evidence.

Only peer-reviewed Persian and English-language studies will be eligible. Grey literature, including conference abstracts, theses, and dissertations, will be excluded to maintain a consistent evidence base comprising studies that have undergone formal peer review. Duplicate publications will also be excluded. Duplicate records will be identified and removed prior to screening. For each database, tailored search strategies will be developed using relevant keywords, controlled vocabulary terms, and synonyms related to NSSI and associated risk and protective factors. The study selection process will be documented using a PRISMA flow diagram.

### Search strategy for electronic databases

Preliminary scoping searches were initially performed to refine the search strategy. Comprehensive search strategies were subsequently developed for each database to include keywords and controlled vocabulary terms covering the following concepts (see Table 1 for example search strategies):

1. Non-suicidal self-injurious behavior (NSSI).
2. Adolescent populations aged 10–19 years.
3. Association terms (e.g., relationship, risk factor, correlation, protective factor).
4. Study designs.
5. Source type (peer-reviewed primary research).
6. Time frame (studies published from 2015 onward).
7. Language (English and Persian).

**Table 1:** Example search strategies for English language (PsycINFO) and Persian (Magiran) databases.

Database	Search strategy
**PsycINFO (EBSCOhost)**	((DE “Nonsuicidal Self-Injury” OR DE “Head Banging” OR DE “Self-Inflicted Wounds” OR DE “Self-Poisoning”) OR DE “Excoriation Disorder” OR (XB(“self-harm*” OR “self injur*” OR “parasuicid*” OR “self-destructive behavio*” OR “self mutilat*” OR skin pick* OR excoriation* OR self-inflicted OR selfinjur* OR self-immolation OR (self AND (harm* OR injur* OR violenc*)))) OR (KW(“self-harm*” OR “self injur*” OR “parasuicid*” OR “self-destructive behavio*” OR “self mutilat*” OR skin pick* OR excoriation* OR self-inflicted OR selfinjur* OR self-immolation OR (self AND (harm* OR injur* OR violenc*))))) AND (AF(Iran) OR KW(Iran* OR Persia*) OR XB(Iran* OR Persia*))
**Magiran**	“ خودزنی غیرخودکشی یا خودآسیبی غیرخودکشی یا خودجرحی یا خودزنی یا خودآسیبی یا خودجرحی غیرخودکشی”

To address linguistic variations, truncations and wildcards will be employed where appropriate. The list of keywords and thesaurus terms was informed by previous systematic reviews on self-injurious behavior and adolescent mental health, and was refined through discussion with experts in adolescent non-suicidal self-injury research.

### Reference selection

All retrieved records will be imported into Covidence for study management. Duplicate records will be identified and removed using a combination of automated and manual procedures within the platform.

Study selection will be conducted in two stages. First, titles and abstracts will be screened against the eligibility criteria by one reviewer. Second, full-text articles of potentially eligible studies will be independently assessed by two reviewers. Screening will be guided by a prespecified screening protocol detailing the inclusion and exclusion criteria. Discrepancies at any stage of the screening process will be resolved through discussion or, if necessary, consultation with a third reviewer. Reasons for exclusion at the full-text stage will be recorded. The anticipated study selection process is presented in a PRISMA flow diagram (see Fig 1). In the final diagram created after completion of the review, we will report the numbers of records identified, screened, excluded, assessed for eligibility, and ultimately included in the systematic review.

**Fig 1:**
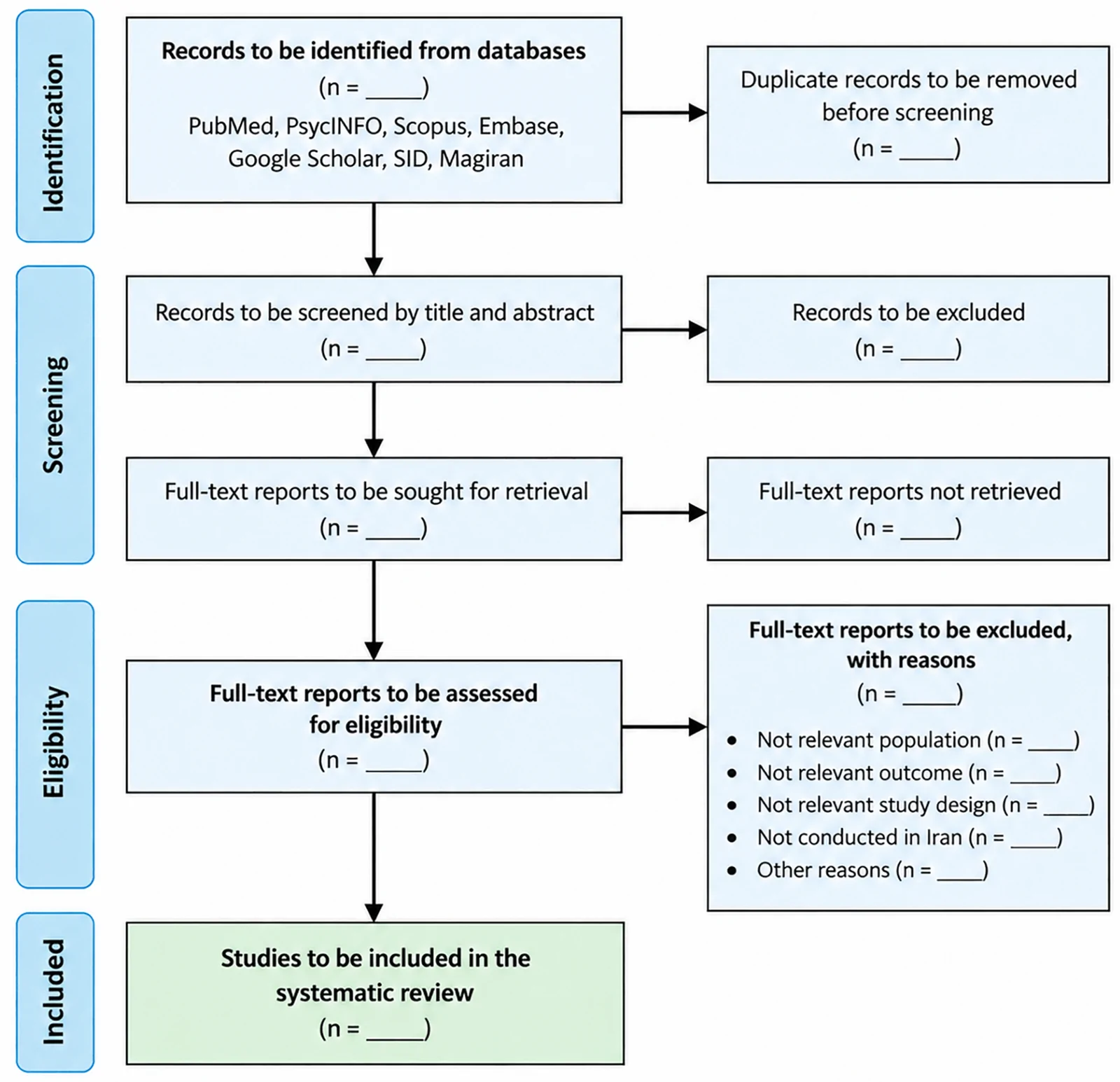
Anticipated PRISMA flow diagram for the planned study identification and selection process.

### Data extraction

For data extraction, a standardized form provided by Covidence will be used. One reviewer will extract data, and the second reviewer will verify its accuracy and completeness. Extracted data will include: study characteristics (title, authors, country/ setting, year of publication, language, period of data collection, keywords), study design or analysis, moderators, mediators, sample size, key findings, and research gaps or future directions identified by the publication’s authors.

### Quality assessment

Using the Mixed Methods Assessment Tool (MMAT) [49] the quality of the included articles will be assessed. This tool specifies criteria such as the appropriateness of data collection methods in qualitative studies, the control of confounders and completeness of data in quantitative studies, and the degree of integration between qualitative and quantitative components in mixed-methods designs. The same procedure as in the screening will be applied: The first author will critically appraise the quality of all included articles, and a second reviewer will perform an assessment on 25% (randomly chosen) of these previously assessed articles. Each study will be evaluated using the relevant domain according to study design and rated as ‘Yes’, ‘No’, or ‘Can’t tell’.

### Data presentation and analysis

We will present and synthesize the data narratively. A descriptive summary table will provide information about study characteristics and methodology of the included research. Associations between risk and protective factors and NSSI outcomes will be organized by outcome categories, and within each outcome group, factors will be clustered into conceptually similar categories informed by clinically meaningful classifications from prior literature, for example, existing psychopathology (internalizing, externalizing and general psychopathology), family relationships (family dynamics, parenting styles and familial resources), demographic variables (socioeconomic status, ethnicity, sexual orientation and gender identity), or peer relationships (bullying, victimization, ostracism and social media interactions). The narrative synthesis will examine the consistency and variability of associations across categories. Where sufficient information is available, we will consider findings descriptively in relation to whether data were collected before, during, or after the pandemic.

## Discussion

This systematic review aims to identify, synthesize, and critically evaluate evidence on risk and protective factors associated with non-suicidal self-injury (NSSI) among adolescents in Iran. By integrating findings across studies, the review seeks to provide a structured overview of factors that may increase vulnerability to, or protect against, NSSI within this sociocultural context. These insights may ultimately inform future prevention and intervention efforts, and improve adolescent mental health.

Several methodological and conceptual challenges are anticipated. First, substantial heterogeneity is expected across studies with respect to design, sampling strategies, measurement of NSSI, and operationalization of risk and protective factors. NSSI may be assessed using self-report questionnaires, clinical interviews, or medical records, each with different implications for reliability, validity, and comparability. Similarly, psychological and contextual variables that will be central to the review, such as psychiatric symptoms, family functioning, or social support, are often measured using diverse instruments or non-standardized indicators. In light of such variations, a narrative synthesis approach, rather than quantitative analysis of effect sizes, is warranted. Second, variability in terminology and conceptual boundaries surrounding self-injurious behaviours represent an additional challenge. Although we will utilize a clear definition of NSSI as self-inflicted injury without suicidal intent, such variability may contribute to misclassification or overlap with broader self-harm constructs, requiring careful screening of the literature. Finally, restricting the review to peer-reviewed studies may increase the risk of publication bias by excluding relevant findings reported in grey literature. However, this restriction will ensure a more consistent evidence base comprising studies that have undergone formal peer review.

Despite these challenges, this review is expected to yield important insights. By systematically organizing evidence across different domains, the review will clarify which risk and protective factors have been most consistently associated with adolescent NSSI in Iran. Furthermore, it also allows us to identify where evidence remains limited or fragmented. Particular attention will be given to protective factors, which are comparatively underrepresented in the Iranian literature but are crucial for informing prevention and early intervention strategies.

The focus on the Iranian context is a key strength of this review. Adolescents in Iran are exposed to a unique constellation of social, economic, and structural stressors, and understanding how these contextual factors intersect with individual vulnerabilities and protective resources is essential for developing culturally appropriate and context-sensitive interventions. The inclusion of both English- and Persian-language studies will broaden the evidence base and allow for greater consideration of context-specific risk and protective factors. Further, applying a consistent operational definition of NSSI will help maintain conceptual clarity and comparability across studies.

### Strengths and limitations of this study

1. Examining all identified risk and protective factors for adolescent NSSI within a single systematic review will allow for meaningful comparisons of the relative importance of each factor.
2. Focusing on the Iranian context enables a context-sensitive examination of how unique structural, social, and economic stressors intersect with individual vulnerabilities and protective resources among adolescents.
3. The review places particular emphasis on protective factors, an area that has received limited attention in Iranian research but is critical for guiding prevention and early intervention strategies.
4. By limiting the review to studies published since 2015, we aim to capture contemporary evidence on risk and protective factors relevant to Iranian adolescents’ current social and cultural context. If sufficient information is available, the review will consider findings in relation to data that were collected before, during, or after the Covid-19 pandemic.
5. Restricting the review to studies that are peer-reviewed may increase the risk of publication bias due to exclusion of findings reported in grey literature.

## Supporting information

S1 File PRISMA-P checklist. (DOCX)
